# Resemblance and differences in dietary restriction nephroprotective mechanisms in young and old rats

**DOI:** 10.18632/aging.103960

**Published:** 2020-09-24

**Authors:** Nadezda V. Andrianova, Ljubava D. Zorova, Irina B. Pevzner, Vasily A. Popkov, Valery P. Chernikov, Denis N. Silachev, Egor Y. Plotnikov, Dmitry B. Zorov

**Affiliations:** 1 A.N. Belozersky Institute of Physico-Chemical Biology, Lomonosov Moscow State University, Moscow 119992, Russia; 2 Faculty of Bioengineering and Bioinformatics, Lomonosov Moscow State University, Moscow 119992, Russia; 3 V.I. Kulakov National Medical Research Center of Obstetrics, Gynecology and Perinatology, Moscow 117997, Russia; 4 Research Institute of Human Morphology, Moscow 117418, Russia; 5 Sechenov First Moscow State Medical University, Institute of Molecular Medicine, Moscow 119991, Russia

**Keywords:** aging, caloric restriction, kidney injury, ischemia/reperfusion, mitochondria

## Abstract

Dietary restriction (DR) is the strategy ameliorating the morbidity of various pathologies, including age-associated diseases. Acute kidney injury (AKI) remains a problem for the elderly with DR being a promising approach for diminishing its consequences. We evaluated the possible nephroprotective potential of short-term DR in young and old rats. DR in young rats resulted in pronounced beneficial effects normalizing lipid metabolism (triglycerides concentration, adiponectin level) activating autophagic-lysosomal system evaluated by LC3II/LC3I ratio, LAMP1, p62/SQSTM1 levels, and LysoTracker Green staining. DR had a remarkable recovering effect on mitochondrial structure and functions including regaining of mitochondrial membrane potential, the elevation of SIRT-3, PGC-1α, _Bcl-XL_ levels and partial restoration of ultrastructure. The beneficial effects of DR resulted in the mitigation of oxidative stress including a decrease in levels of protein carbonylation and lipid peroxidation. Aging led to decreased activity of autophagy, elevated oxidative stress and impaired kidney regenerative capacity. Eventually, in old rats, even 8-week DR was not able to ameliorate AKI, but it caused some rejuvenating effects including elevation of mitochondrial membrane potential and Bcl-X_L_ levels, as well as lowered severity of the oxidative stress. Thus, the age-associated decline of protective signaling demands extended DR to achieve nephroprotective potential in old animals.

## INTRODUCTION

Caloric or dietary restriction (DR) is one of the most efficient approaches to prevent aging and ameliorate different pathological conditions including age-associated diseases [1, 2]. DR is defined as a reduction of food consumption with no obvious signs of malnutrition [3]. During DR, multiple signaling pathways are activated including those affecting growth regulation, metabolism, autophagy, response to oxidative stress, regeneration, and inflammation [4]. Ultimately, based on elucidated mechanisms, DR was suggested to be a promising approach for the treatment of various age-associated diseases [5].

One of the most pronounced age-associated diseases is acute kidney injury (AKI), which affects about 20% of all hospitalized patients in developed countries [6], predominately people over 65 years [7]. AKI is a clinical syndrome characterized by a sudden decline in renal function [8] which does not have specific treatment and has a very high mortality rate due to its transition to chronic kidney disease (CKD) and end-stage renal disease [9]. The majority of cases of AKI are caused by ischemia [10], and DR was proven to diminish ischemic injuries in almost all organs [11–14] including kidneys [15, 16].

However, the majority of studies on the nephroprotective effects of DR use young animals as model organisms [15–19], although patients with AKI are mostly elderly [20]. In the process of aging, renal tissue, as well as other tissues, becomes more sensitive to various damaging factors reflecting progressive molecular, structural, and functional deleterious changes [21]. Cells of all organs acquire some age-related changes such as cell cycle arrest, senescence-associated secretory phenotype, mitochondrial dysfunction, etc [22–24]. As a result, approaches proven to be efficient in young animals, do not always manifest their protective effects in old organisms [25, 26].

The additional question arises about the optimal duration of DR exposure [27]. The long-term DR, commensurable with lifespan, is admitted to afford the most beneficial effects [3]. Nevertheless, even short-term episodes of DR are shown to result in similar positive effects [28, 29]. In our previous study, we reported that DR for 4 weeks possessed a remarkable nephroprotective effect observed only in young rats, i.e., the same duration of DR in old rats did not exert any significant positive impact [30].

In this study, we subjected old rats to a more prolonged DR protocol that lasted for 8 weeks. We analyzed the nephroprotective potential of DR in young and old rats and elucidated the mechanisms of DR tracking its beneficial effects on animals of different ages. In young and old rats, we studied the systemic influence of DR, analyzed the proliferative capacity of kidney tissue in response to damage, and evaluated the functioning of the autophagic-lysosomal system and mitochondria and the severity of oxidative stress.

## RESULTS

### Systemic effects of dietary restriction

**Figure 1:**
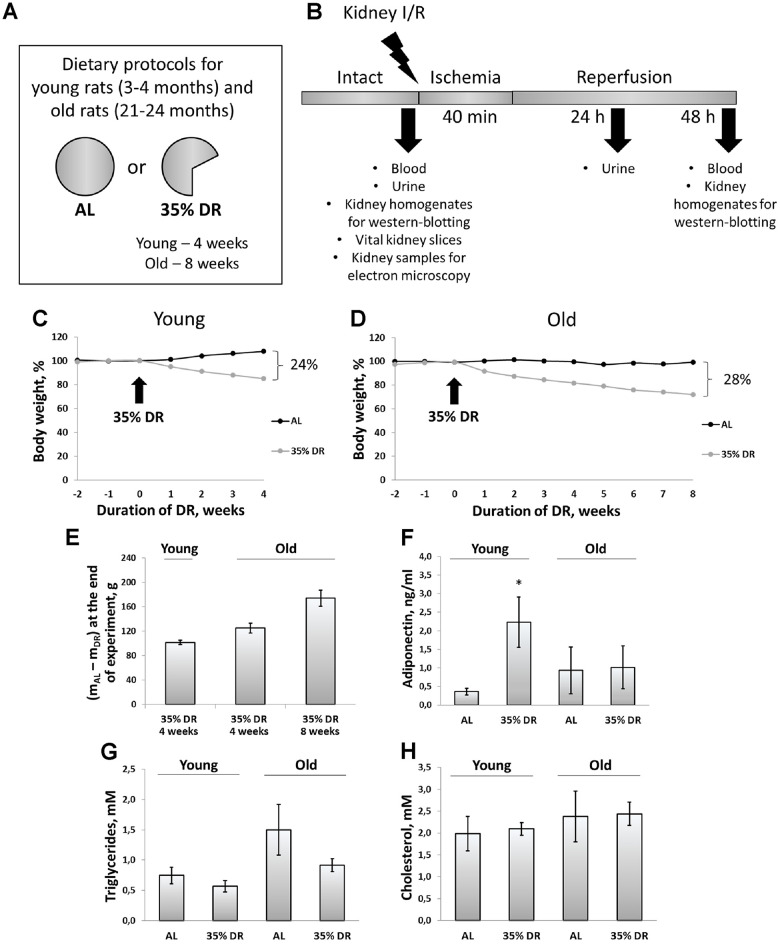
**Experimental design and systemic effects of DR.** (**A**) Dietary protocols for young and old rats during the experiment; (**B**) Experimental design; (**C**) Weight changes in young rats (n=12 for each group); (**D**) Weight changes in old rats (n=12 for each group); (**E**) The difference in body weight between AL group and DR group after 4 weeks and 8 weeks from the start of DR; (**F**) Adiponectin levels in serum of young and old rats on AL and 35% DR diet; (**G**) Triglycerides concentrations in serum of young and old rats on AL and 35% DR diet; (**H**) Total cholesterol concentrations in serum of young and old rats on AL and 35% DR diet. *p < 0.05 compared to young AL-group. N=5 for each group in (**F**–**H**). AL, ad libitum, DR, dietary restriction, I/R ischemia/reperfusion.

During 4 weeks, young *ad libitum* (AL) rats on average gained 36 g (8% from their initial weight), whereas rats subjected to DR lost 15% of their initial weight (which amounted to 66 g) (Figure 1C). At the end of the experiment, the total difference of body mass between AL rats and rats on DR was 23%, which corresponded to 102 g (Figure 1E). During the study, old control rats kept on AL diet did not gain or lose any weight. In old rats, 8 weeks of DR caused pronounced weight loss (28%) (Figure 1D). For the first 4 weeks of DR, old rats lost 125 g on the average, whereas after 8 weeks body weight decreased by 174 g (Figure 1E).

In experimental animals, we also analyzed changes in lipid metabolism and its regulation. First, we measured adiponectin concentrations in the serum of rats exposed to AL diet or DR (Figure 1F). Adiponectin is a hormone secreted by adipose tissue (adipokine) which stimulates fatty acid oxidation and inhibits glucose production resulting in an improvement in whole-body energy homeostasis [31]. The measurement of adiponectin concentration using ELISA revealed more than a 6-fold increase in adiponectin levels in the serum of rats exposed to DR vs AL rats (from 0.36 ± 0.09 ng/ml to 2.23 ± 0.67 ng/ml). Serum of old AL rats contained higher adiponectin than serum of young AL rats. However, DR in old rats did not cause an increase in adiponectin levels observed in young animals.

Accordingly, the serum of old AL rats had two times higher concentrations of triglycerides of young AL animals (Figure 1G). DR in both age groups led to a drop in triglycerides levels. Total cholesterol concentration in the serum did not change in response to DR or aging (Figure 1H).

Of note, we analyzed the extent of renal fibrosis in all groups of rats and revealed that the level of α-smooth muscle actin in kidneys from old rats was 2.7 times higher than in kidneys from young rats (Supplementary Figure 1). DR for 8 weeks did not decrease the extent of fibrosis in old rats.

### Effect of DR on the severity of AKI

**Figure 2:**
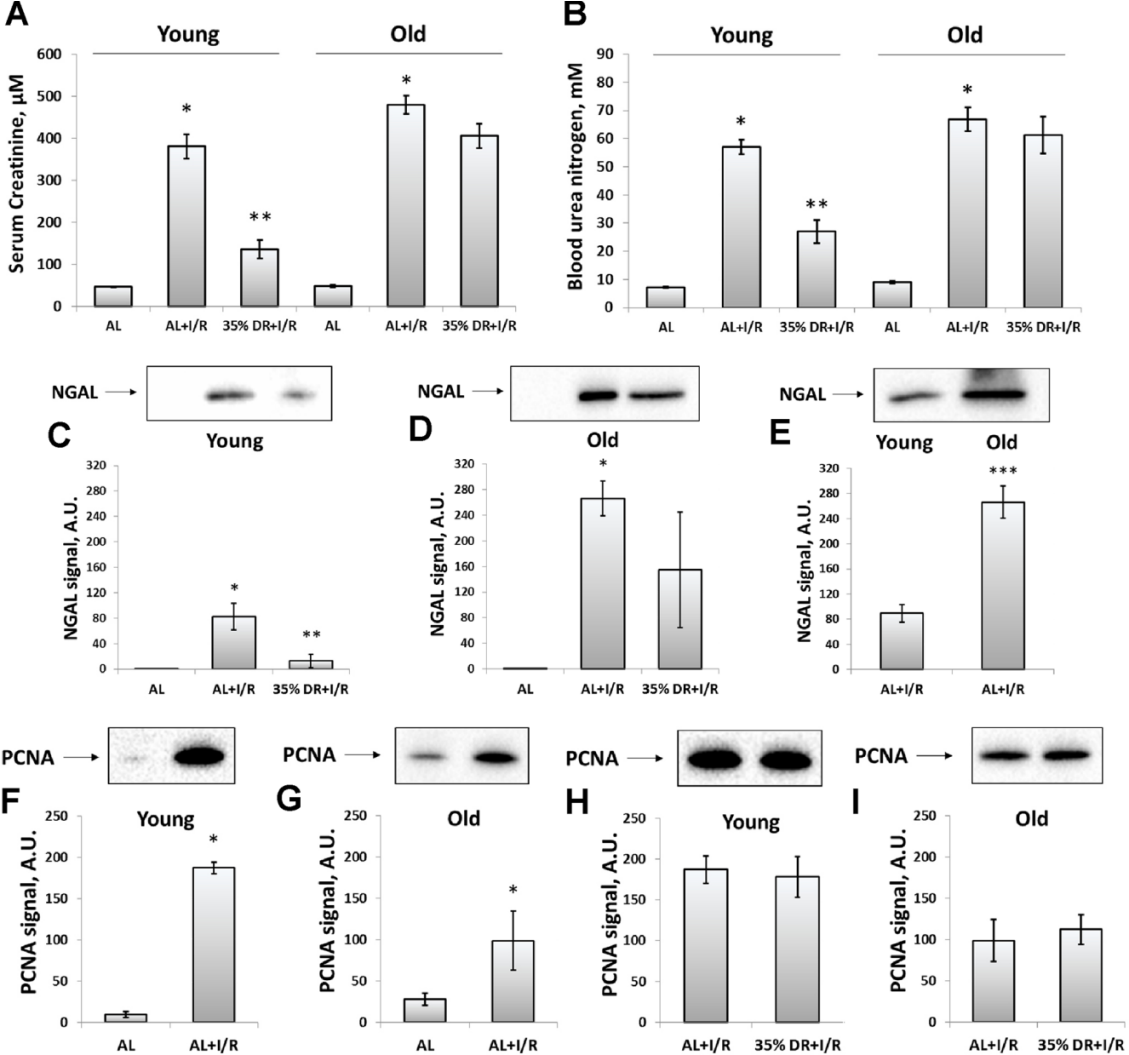
**Assessment of renal function after I/R in young and old rats with or without DR exposure.** (**A**) The severity of AKI evaluated by serum creatinine levels 48 h after I/R; (**B**) The severity of AKI evaluated by blood urea nitrogen levels in serum 48 h after I/R; (**C**) The renal tissue injury evaluated by NGAL levels in urine 24 h after I/R in young rats; (**D**) The renal tissue injury evaluated by NGAL levels in urine 24 h after I/R in old rats; (**E**) The comparison of NGAL levels in the urine of young and old rats 24 h after I/R; (**F**, **G**) The increase of proliferation evaluated by PCNA levels in kidney tissue 48 h after I/R in young and old rats; (**H**, **I**) PCNA levels in kidney tissue 48 h after I/R in young and old rats exposed to DR diet. *p < 0.05 compared to AL-group, **p < 0.05 compared to AL+I/R-group, ***p < 0.05 compared to young AL+I/R-group. For young AL rats n=5, for young DR rats n=6, for old AL rats n=6, for old DR rats n=6. AL, ad libitum, DR, dietary restriction, I/R, ischemia/reperfusion.

Since ischemic injury is the most common factor leading to AKI [10], we used kidney ischemia/reperfusion (I/R) as a model evaluating AKI severity by measuring serum creatinine (SCr) and blood urea nitrogen (BUN) levels. In both young and old rats, we observed a more than 8-fold increase in SCr and BUN 48 h after renal I/R, which indicated the onset of AKI (Figure 2A, 2B). DR caused less severe injury, as evidenced by a less pronounced increase of SCr and BUN levels. SCr concentration dropped from 380.9 ± 28.6 μM in young AL ischemic rats to 136.3 ± 21.7 μM in DR group. Similar changes were observed in BUN levels which decreased from 56.9 ± 2.6 mM to 27.0 ± 4.2 mM in rats kept in DR. However, old rats kept on longer DR (8 weeks) did not show a significant decrease of AKI revealing the loss of a general nephroprotective effect of DR.

In addition to SCr and BUN which are classical indexes mostly reflecting renal functioning, we also analyzed the levels of neutrophil gelatinase-associated lipocalin (NGAL) in urine (Figure 2C–2E). NGAL is suggested to be a biomarker of renal tissue injury which allows to reveal AKI in an early stage and more accurate than SCr and BUN do [32]. Normally, NGAL is absent in the urine and after I/R we found its great increase (Figure 2C, 2D). The levels of urine NGAL were evaluated 24 h after I/R (i.e. 24 h before collecting blood for SCr and BUN) according to its peak concentration [33]. While stayed increased after I/R, NGAL levels dropped 6 times in the urine of young DR rats (Figure 2C). In old rats from DR-group, there was essential heterogeneity in response: some rats showed reduced levels of NGAL, while other animals demonstrated the same rise as old rats from AL-group (Figure 2D). A comparison of NGAL levels in the urine of young and old rats revealed a more pronounced content of this biomarker in old animals exposed to I/R (Figure 2E).

The proliferation in the kidney tissue after injury was assessed by measuring proliferating cell nuclear antigen (PCNA). In young rats exposed to I/R, the PCNA signal increased 19 times compared to intact rats indicating activation of regeneration and great proliferative capacity of kidney cells 48 h after ischemic challenge (Figure 2F). In old rats, PCNA levels also increased, but only 4.5 times which could be the evidence of deterioration of regenerative mechanisms in the aging process (Figure 2G). DR did not increase the proliferative capacity of kidney cells after I/R in animals of both ages (Figure 2H, 2I).

### The activity of the autophagic-lysosomal system

**Figure 3:**
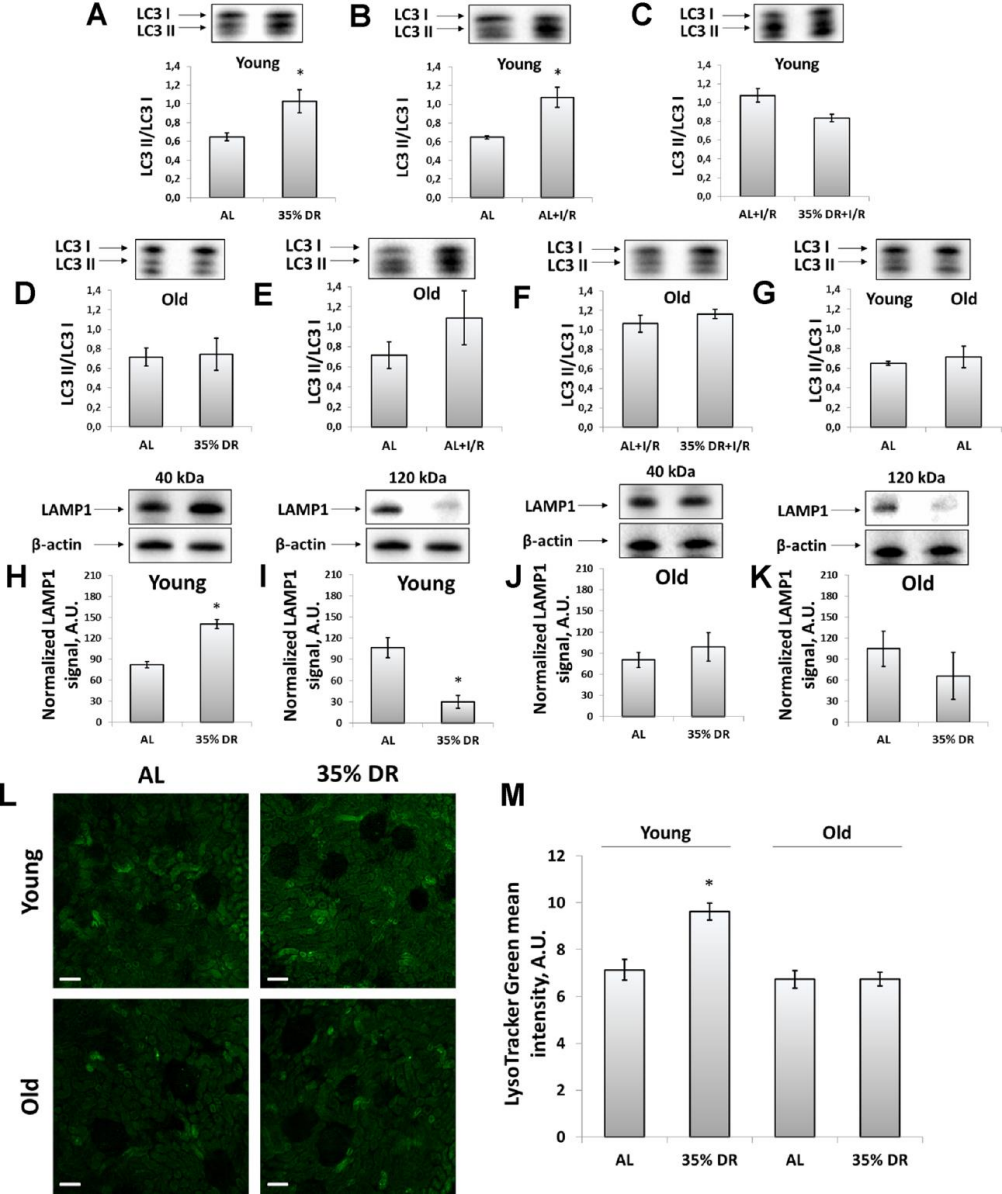
**Assessment of autophagic activity in young and old rats.** (**A**) LC3 II/LC3 I ratio in kidney tissue of young rats in response to DR; (**B**) LC3 II/LC3 I ratio in kidney tissue of young rats in response to I/R; (**C**) The comparison of LC3 II/LC3 I ratio in kidney tissue of young rats after I/R with or without DR diet; (**D**) LC3 II/LC3 I ratio in kidney tissue of old rats in response to DR; (**E**) LC3 II/LC3 I ratio in kidney tissue of old rats in response to I/R; (**F**) The comparison of LC3 II/LC3 I ratio in kidney tissue of old rats after I/R with or without DR diet; (**G**) Comparison of LC3 II/LC3 I ratio in kidney tissue of young and old rats; (**H**, **I**) Levels of non-glycosylated (40 kDa) and glycosylated (120 kDa) forms of LAMP1 in kidney tissue of young rats; (**J**, **K**) Levels of non-glycosylated (40 kDa) and glycosylated (120 kDa) forms of LAMP1 in kidney tissue of old rats; (**L**) Lysosomes staining with LysoTracker Green probe revealed by confocal microscopy of vital kidney slices. Scale bar, 100 μm; (**M**) Quantification of LysoTracker Green fluorescence intensity. *p < 0.05 compared to AL-group. For young AL rats n=5, for young DR rats n=6, for old AL rats n=6, for old DR rats n=5. AL, ad libitum, DR, dietary restriction, I/R, ischemia/reperfusion.

We analyzed the levels of light chain 3 (LC3) protein to address the activation of autophagy kidney in response to DR and I/R. LC3 II/LC3 I ratio is a well-known marker of the number of autophagosomes and its increase reflects the activation of autophagy [34]. Young rats demonstrated significant activation autophagic process in response to both DR and I/R (Figure 3A, 3B). After DR, I/R did not cause an additional rise of LC3 II/LC3 I ratio (Figure 3C). In old animals, there was no significant increase in LC3 II/LC3 I ratio (Figure 3D, 3E) and DR was not potent in amelioration of the deterioration (Figure 3F). However, the LC3 II/LC3 I ratio in kidneys of old AL rats did not change compared to young AL animals (Figure 3G).

Since autophagy is tightly coupled to the lysosomes maintenance, we assessed the levels of lysosomal-associated membrane protein 1 (LAMP1), which is one of the major lysosomal proteins [35]. Using western-blotting, we could observe different LAMP1 isoforms: the band corresponding to 40 kDa represented a core non-N-glycosylated protein while the band near 120 kDa represented a glycosylated form of LAMP1 [36]. We found that in response to DR, the intensity of the 40 kDa band was increased indicating the elevation of the total lysosome content in the renal tissue of rats (Figure 3H). Simultaneously, in the kidney samples of young rats, we observed the reduction of 120 kDa isoform in response to DR reflecting a drop of the glycosylated form of LAMP1 (Figure 3I). Kidneys of old rats retained the same trend, but to a lesser extent and with higher heterogeneity (Figure 3J, 3K).

We confirmed DR-induced alterations in lysosomes content by staining vital kidney slices with LysoTracker Green dye designed to selectively stain acidic compartments, such as lysosomes and autophagolysosomes [34]. DR performed on young rats resulted in elevated fluorescence of LysoTracker Green, that could be interpreted as an increase of lysosomes amount. Old rats subjected to 8-week DR did not show such a lysosomal increase. Furthermore, LysoTracker Green did not show a significant difference between unexposed young and old AL rats (Figure 3L, 3M).

In addition, we have measured the levels of p62/SQSTM1, which is a ubiquitin-binding protein that attaches ubiquitinated proteins to the adapter proteins while autophagofore formation and thus promoting their degradation in the autophagolysosomes [37]. We found that in young animals DR caused the significant drop of p62/SQSTM1 levels indicating the increased autophagic flux and activation of autophagy, whereas in old rats DR had a weak effect on p62/SQSTM1 levels (Supplementary Figure 2).

### Effects of the DR on mitochondria-associated pathways

**Figure 4:**
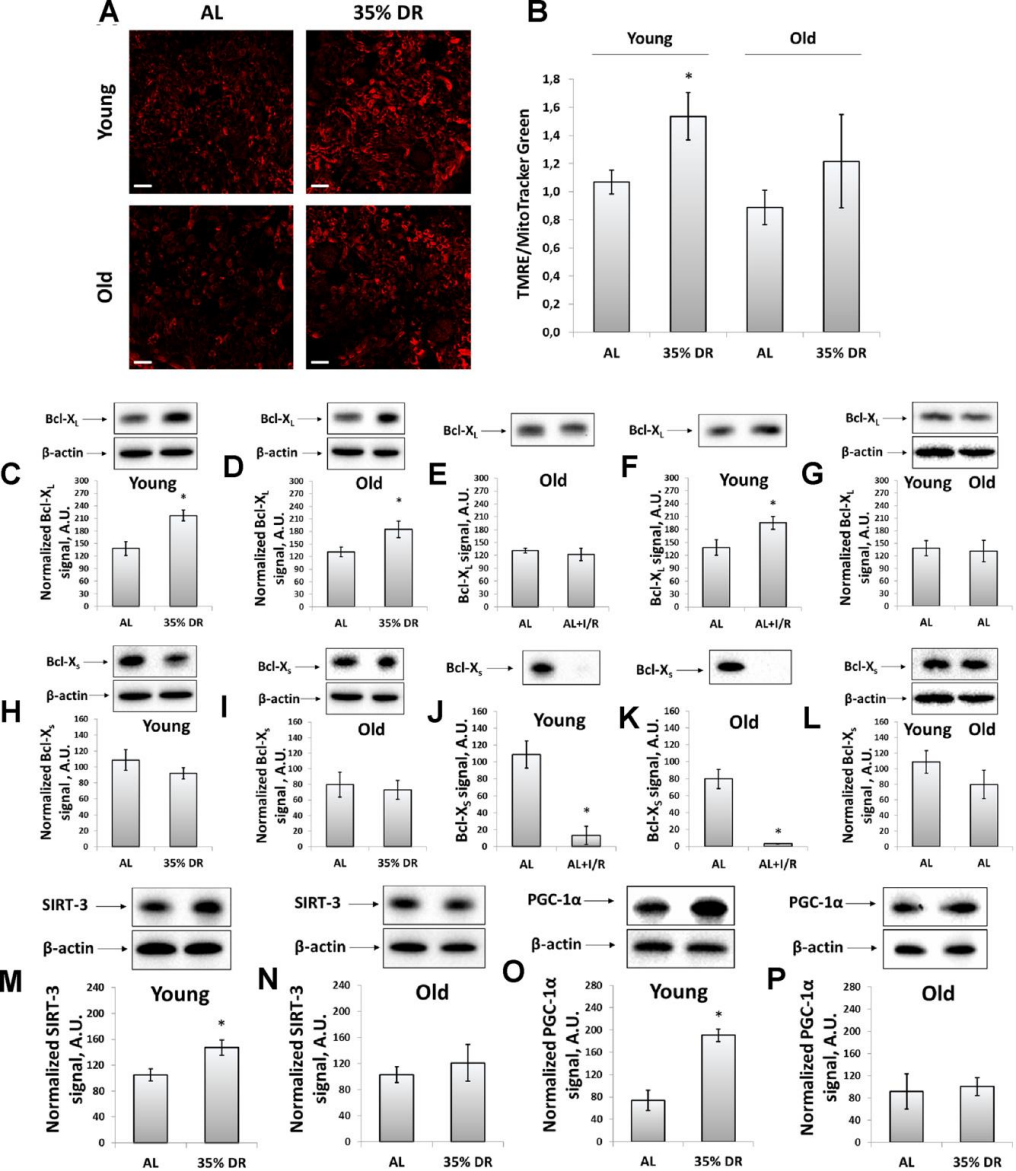
**Evaluation of the mitochondrial potential, anti-apoptotic and pro-apoptotic proteins, the intensity of mitochondrial biogenesis, and the levels of mitochondrial deacetylase SIRT-3.** (**A**) Confocal microscopy of vital kidney slices loaded with mitochondrial probe TMRE. Scale bar, 100 μm; (**B**) Mitochondrial membrane potential estimated by TMRE fluorescence intensity normalized to MitoTracker Green fluorescence intensity; (**C**, **D**) Levels of Bcl-X_L_ in kidney tissue of young and old rats after DR; (**E**, **F**) Levels of Bcl-X_L_ in kidneys of young and old rats in response to I/R; (**G**) Comparison of Bcl-XL levels in kidney tissue of young and old rats; (**H**, **I**) Levels of Bcl-X_s_ in kidney tissue of young and old rats after the DR; (**J**, **K**) Levels of Bcl-X_s_ in kidney tissue of young and old rats in response to I/R; (**L**) Comparison of Bcl-XS levels in kidney tissue of young and old rats; (**M**, **N**) Levels of SIRT-3 in kidney tissue of young and old rats after the DR; (**O**, **P**) Levels of PGC-1α in kidney tissue of young and old rats after the DR. *p < 0.05 compared to AL-group. For young AL rats n=5, for young DR rats n=6, for old AL rats n=6, for old DR rats n=5. AL, ad libitum, DR, dietary restriction, IR, ischemia/reperfusion.

The transmembrane potential was assessed using the staining of vital kidney slices with tetramethylrhodamine ethyl ester (TMRE) dye (Figure 4A). TMRE is proved to accumulate in normally functioning mitochondria following their membrane potential [38]. The mean TMRE fluorescence intensity was normalized to MitoTracker Green fluorescence intensity since MitoTracker Green labels mitochondria in a manner that is independent on the membrane potential [39]. We observed that DR caused higher TMRE accumulation in kidney cells of both young and old rats indicating an increase of mitochondrial fraction with higher membrane potential (Figure 4A, 4B). The mean MitoTracker Green fluorescence intensity in vital kidney slices from old rats tended to be higher than in young animals (Supplementary Figure 3).

We also analyzed mitochondrial status by measuring the levels of Bcl-2 family proteins, anti-apoptotic Bcl-X_L_ and pro-apoptotic Bcl-X_S_ [40]. Detection of these proteins was evaluated using the same antibodies since Bcl-X_L_ and Bcl-X_S_ share most of the protein domains being different in molecular weights [41]. DR resulted in a 57% rise in Bcl-X_L_ content in young rats (Figure 4C), as well as led to a 40% increase in its levels in old animals (Figure 4D). In young animals exposed to I/R, a slight increase in Bcl-X_L_ levels was found (Figure 4E), which was not observed in kidneys of old rats (Figure 4F). The Bcl-X_S_ levels did not alter after DR in rats of both ages (Figure 4H, 4I), however, I/R caused a multifold drop in Bcl-X_S_ content (Figure 4J, 4K). Aging did not affect the levels of Bcl-X_L_ and Bcl-X_S_ (Figure 4G, 4L).

In addition, in kidney homogenates, we measured the content of mitochondrial deacetylase SIRT-3, which is believed to be a mitochondrial stress sensor that can modulate the activity of mitochondrial proteins involved in metabolism and oxidative stress regulatory pathways [42]. In young rats, we observed a significant increase in SIRT-3 levels caused by DR (Figure 4M), whereas in kidneys of old animals we did not find any DR-induced alterations in SIRT-3 levels (Figure 4N).

We evaluated mitochondrial biogenesis through evaluation of peroxisome proliferator-activated receptor gamma coactivator 1-alpha (PGC-1α) levels, which is known to play an essential role in metabolic reprogramming in dietary interventions coordinating the expression of genes involved in glucose and fatty acids metabolism [43]. Indeed, in kidneys of young rats exposed to DR, we observed more than 2.5 times increase in PGC-1α content (Figure 4O), in contrast to old rats having no such DR-induced alterations (Figure 4P). Thus, we concluded that DR enhanced mitochondrial biogenesis in the kidney of young but not of old rats.

**Figure 5:**
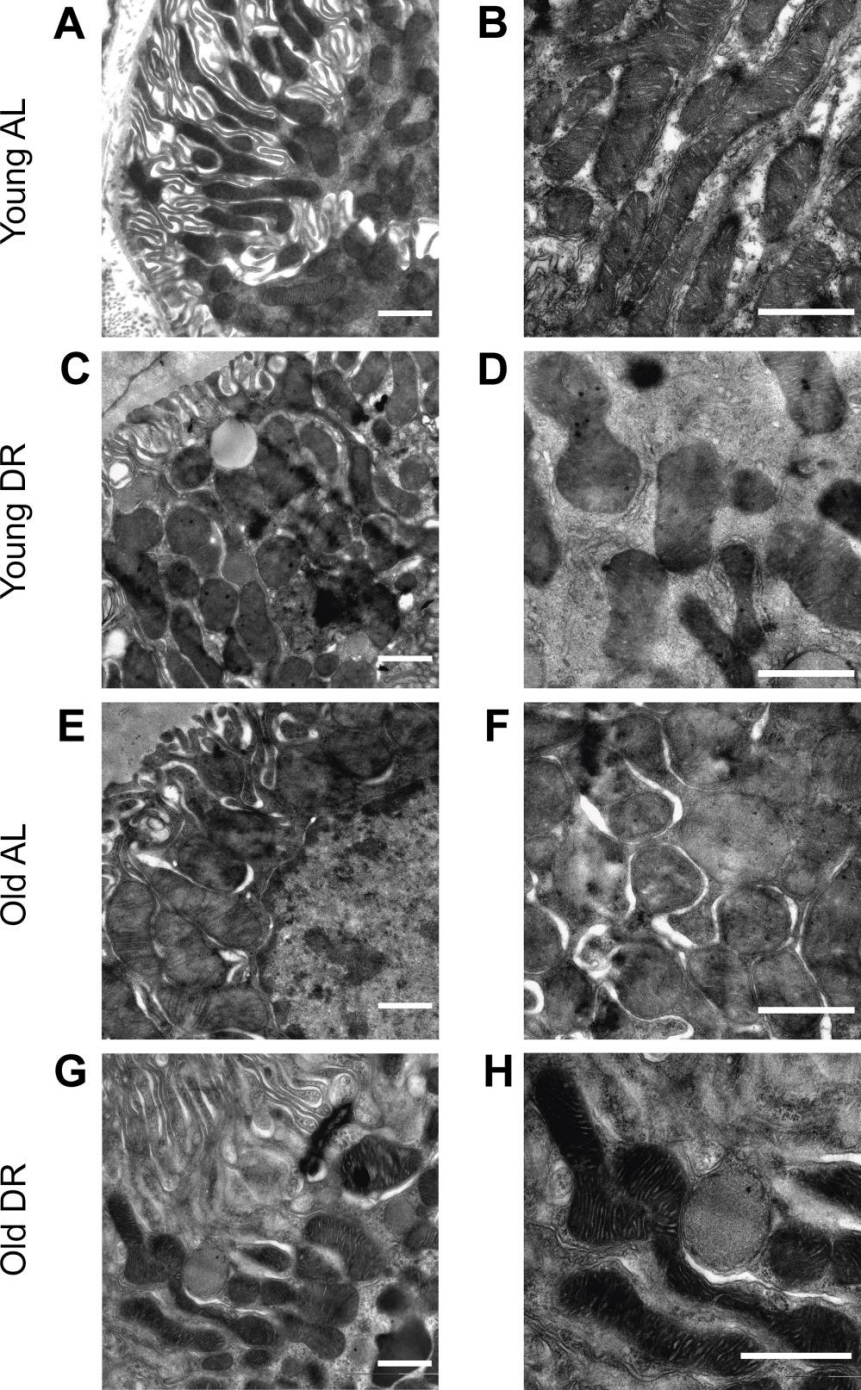
Ultrastructure of mitochondria of renal tubular cells in the intact kidney of young (**A**, **B**) and old (**E**, **F**) rats and those after DR (**C**, **D** and **G**, **H**, correspondingly). Renal tubular cells in young rats contain mitochondria in the orthodox configuration, while mitochondria in the old kidney demonstrate slight swelling of intercristae space and matrix. Tubular epithelium of kidney after DR exposure demonstrates noticeable condensation of the mitochondrial matrix in both young (**C**, **D**) and old (**G**, **H**) rats. Scale bar, 1 μm. For young AL rats n=5, for young DR rats n=6, for old AL rats n=6, for old DR rats n=5. AL, ad libitum, DR, dietary restriction.

The analysis of mitochondrial ultrastructure was conducted using transmission electron microscopy (Figure 5). Mitochondria in the renal tubular cells of young animals had characteristic features with pronounced outlines of mitochondrial profiles and with a quite strict parallel orientation of cristae and no obvious signs of swelling (Figure 5A, 5B). Local extensions of the intracristae space were obvious. The mitochondria of the tubular cells of old rats were more enlightened, which indicates a relative swelling of the matrix, which is often accompanied by partial de-energization (Figure 5E, 5F). At the same time, the parallel arrangement of cristae observed in young rats was less pronounced, that is, the cristae were oriented more chaotically. When DR was imposed to animals, both in the case of young (Figure 5C, 5D) and old rats (Figure 5G, 5H), there was a noticeable condensation of the mitochondrial matrix which is a sign of a decrease in the respiratory activity of the mitochondria often caused by increased membrane potential. In older animals exposed to DR, the mitochondrial cristae begin to be oriented more parallel to each other.

### ROS production and oxidative stress

**Figure 6:**
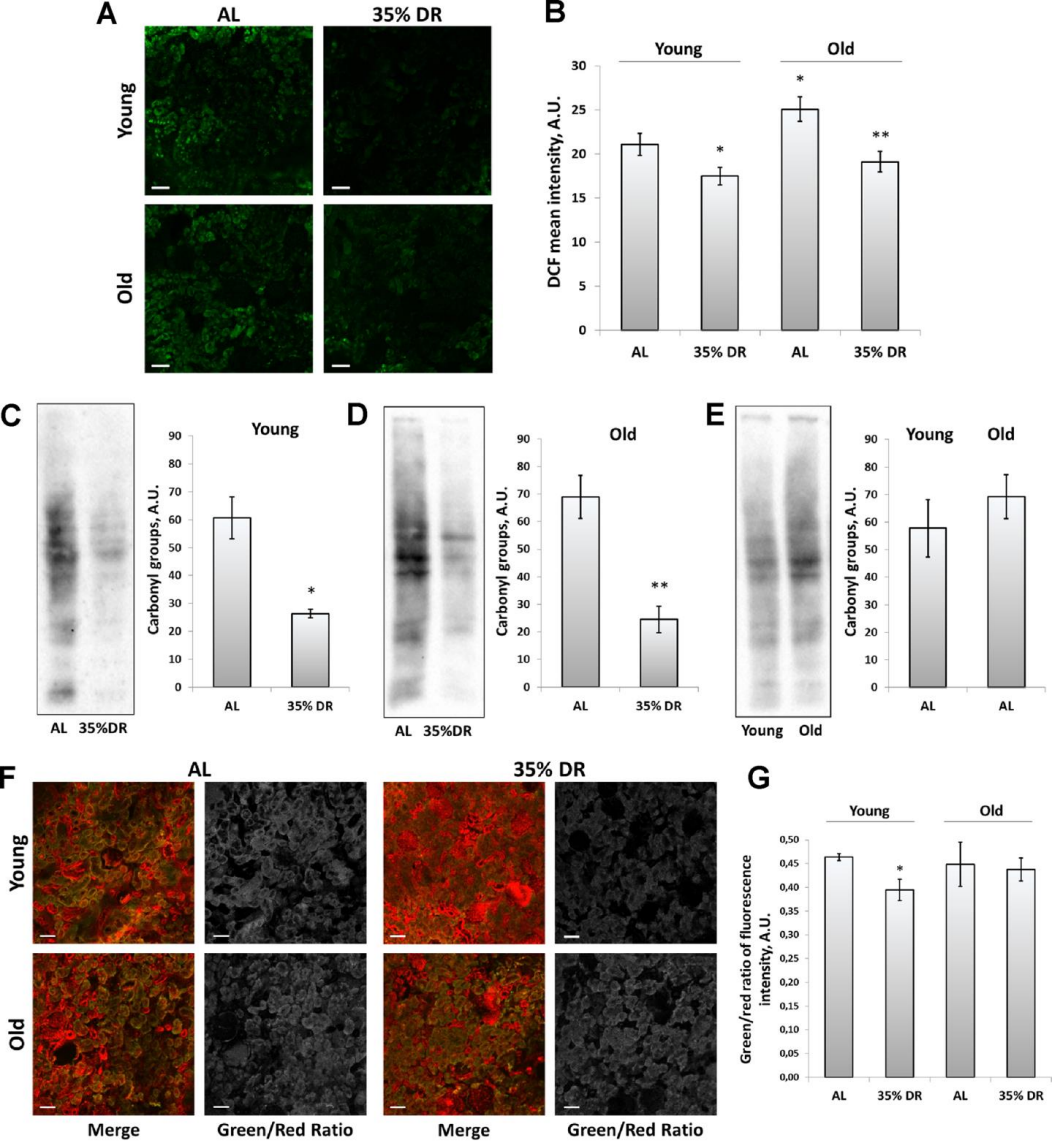
**ROS production and oxidative stress in the kidney.** (**A**) Confocal microscopy of DCF-loaded vital kidney slices. Scale bar, 100 μm; (**B**) Quantification of DCF fluorescence intensity on confocal images; (**C**) OxyBlot staining for carbonylated proteins in kidney tissue of young DR rats; (**D**) OxyBlot staining for carbonylated proteins in kidney tissue of old rats after DR; (**E**) Comparison of carbonylated protein levels in kidney tissue of young and old rats; (**F**) Confocal microscopy of vital kidney slices stained with Lipid Peroxidation Kit. Scale bar, 100 μm; (**G**) Estimation of lipid peroxidation measured by a green/red ratio of fluorescence intensity. *p < 0.05 compared to young AL-group, *p < 0.05 compared to old AL-group. For young AL rats n=5, for young DR rats n=6, for old AL rats n=6, for old DR rats n=5. AL, ad libitum, DR, dietary restriction.

To explore the involvement of oxidative stress in the interplay between mechanisms of aging and DR, the assessment of reactive oxygen species (ROS) levels was performed using staining of vital kidney slices with 2’,7’-dichlorodihydrofluorescein diacetate (DCF) (Figure 6A), the probe for ROS [44]. We found that old rats’ kidneys acquired a more intensive DCF signal than the young kidney (Figure 6A, 6B). DR significantly reduced mean DCF fluorescence intensity both in young and old animals (Figure 6A, 6B).

To assess the contribution of other factors of oxidative stress, we also analyzed total protein carbonylation by OxyBlot Protein Oxidation Detection Kit. Protein carbonylation is a result of the oxidative modification of proteins by ROS and reactive carbonyls which often leads to protein malfunction [45]. We found that DR exposed to 4 weeks in young or 8 weeks in old rats resulted in a great drop in carbonylated proteins (Figure 6C, 6D). The levels of protein carbonylation tended to be higher in kidneys of old rats (Figure 6E).

In addition, we estimated the intensity of lipid peroxidation using the Lipid Peroxidation Kit based on the oxidation of BODIPY® 581/591 dye (Figure 6F). This dye is accumulated in membranes and upon oxidation displays a shift in peak fluorescence emission from 590 nm to 510 nm [46]. The ratio between green and red channels is believed to reflect the levels of lipid peroxidation. Our analysis revealed the decrease in green to red channels ratio in kidney slices from DR rats, but only in young animals (Figure 6G).

## DISCUSSION

In this study, we explored the nephroprotective potential of DR in young and old rats to gain insight into the mechanisms that mediated the beneficial effects of DR in animals of different ages. Previously, we were working with 4-week DR imposed on both young and old rats and evaluated that in old animals this protocol was not efficient enough to ameliorate AKI [30]. The question about the duration of DR and its efficiency was repeatedly raised [3, 27], so we tried to narrow this gap by comparing our previous data with more prolonged DR protocol in old rats.

There is no doubt that the greatest effects are observed during long-term DR, which duration is comparable with a lifespan of the exposed subject [3]. Regarding the kidneys, long-term DR delayed the onset and diminished the severity of age-associated renal diseases preventing proteinuria and renal membrane lipid deposition, attenuating age-related prostaglandin imbalances [47–50]. Nevertheless, even short-term episodes of DR are shown to have beneficial effects. In 24-month-old rats, DR lasting for 10 days led to a significant reduction in ROS and lipid peroxides levels, COX-2 activity, and retarded proinflammatory pathways in kidney tissue [28]. Similarly, 4-week DR reduced inflammation, cellular stress, fibrosis, as well as restored the ability of normal cell-cycling, DNA replication, and shifted the genomic profile in kidney tissue of old rats [29].

In this study, we have chosen the 8-week DR protocol for old animals. Since in young rats 4 weeks of DR had a very pronounced nephroprotective effect [30] which was consistent with other studies [15, 51], we did not change the details of this protocol besides its length. The majority of papers dealing with a short-term DR in old rodents used 8 weeks of DR and it resulted in a range of beneficial consequences. For instance, old rats subjected to an 8-week DR showed a lower level of fibrosis, better autophagy functioning, as well as reduced levels of senescence indexes such as p16, p21, and β-galactosidase [52, 53]. The same group reported that such DR protocol was efficient in old rats ameliorating the severity of cisplatin nephrotoxicity [54]. Similar results were observed in other organs. For example, two-months DR caused comparable changes in hepatic gene expression which were observed in lifelong DR [55]. After 2 months of DR with every-other-day feeding of 28-month old rats, the histone carbonylation in the liver was increased to the levels close to that in young animals [56]. Ultimately, 8 weeks of DR initiated at 19 months of age resulted in a significant extension of median and maximum lifespan in mice [57].

In humans, different protocols of DR were suggested and evaluated [58]. First, CALERIE-1 three pilot studies were conducted with the participation of overweight people. The reduction of energy intake was 20%-25% of their normal daily diet and lasted from 6 to 12 months. Such DR protocols resulted in normalization of metabolism, including weight loss, a decrease of serum insulin, improved insulin sensitivity, and a decline in markers of oxidative stress [59–61]. Expression of genes encoding proteins involved in mitochondrial functions such as PGC-1α, eNOS, SIRT-1, and PARL was also increased by DR [62]. In the CALERIE-2 multicenter trial, only young people with normal weight took part, and they were subjected to 25% DR for 2 years [63]. The clinical trial demonstrated that mild DR improved cardiometabolic risk factors, normalized glucose and lipid metabolism, cytokines profile, and C-reactive protein levels [64–66].

In our study, 35% DR for 4 weeks in young rats and 8 weeks in old ones led to a significant weight loss and was also accompanied by normalization of triglycerides levels (Figure 1). In addition, we observed an elevation of adiponectin concentration in the serum of young rats after DR (Figure 1). Such an increase of adiponectin level during DR is considered to be associated with the loss of adipose tissue [67, 68]. Unlike most other adipokines, adiponectin gene expression and blood concentration of the protein are inversely associated with fat mass and obesity [69]. Adiponectin is considered as a key hormone to control body metabolism since it increases fatty acid oxidation and insulin sensitivity [70]. Adiponectin is also a classic anti-inflammatory agent, reducing inflammation by inhibiting macrophage differentiation, switching the macrophage phenotype to an anti-inflammatory state, and decreasing expression of Toll-like receptor 4 [71]. Initial studies demonstrated that intraperitoneal injections of mammalian-produced adiponectin lowered systemic glucose and free fatty acid concentrations in mice [72]. However, aging alters the functions of adipocytes, which leads to alterations in the secretion, synthesis, and function of the adipokines. Furthermore, in older individuals increased adiponectin level is associated with higher mortality [73]. Dysregulation of adiponectin in older individuals may be due to loss of function of circulating adiponectin or to increased inflammatory process [74]. We confirmed earlier results that the serum levels of adiponectin in old rats were not affected by DR [75] (Figure 1).

One of the most important targets of DR is the process of autophagy [76]. It is well-known that DR is a strong inducer of autophagy mostly through Akt and AMPK signaling pathways leading to inhibition of mTOR which negatively regulates autophagophore formation [77]. I/R, like other damaging factors, is also able to activate autophagic signaling pathways due to the necessity of survived cells to remove the defective macromolecules and organelles [78]. We observed a significant activation of autophagy in the kidneys of young rats in response to both DR and I/R (Figure 3 and Supplementary Figure 2). However, the processes of autophagy are gradually deteriorated with age leading to deterioration of the quality control mechanism [79]. We also observed the impairment of autophagy since in the kidneys of old rats, proper activation of autophagy in response to DR was not observed, moreover, I/R caused lower autophagy activation, compared to young animals (Figure 3).

The deterioration of autophagy with aging also applies to the functioning of mitophagy, which is believed to be the only mechanism for elimination of poorly functioning mitochondria [80], and a decline in the work of this system must inevitably lead to accumulation of mitochondria with low membrane potential [81]. Several studies have demonstrated that mitochondria isolated from the tissues of old organisms had lower membrane potential than in young ones [82–84]. Potentially, DR is able to enhance the process of mitophagy eliminating mitochondria carrying low membrane potential [30, 85]. Indeed, at the end of the DR period, we observed the increase of mean TMRE fluorescence intensity indicating higher mitochondrial membrane potential even in old kidneys.

The analysis of mitochondrial ultrastructure in tubular renal cells confirms the partial rejuvenating effect of DR. Specifically, while in young animals, mitochondria have a condensed configuration, in old cells the configuration is shifted to a more orthodox state which can be interpreted as a shift to a state of mitochondria with less membrane potential. The data on direct measurements of the membrane potential using a potential-dependent probe TMRE confirms this conclusion (Figure 4). Moreover, DR imposed on either young or old animals caused the transition to a more condensed state which also can be interpreted as a transition to a more hyperpolarized state. And again, fluorescent microscopy of renal sections loaded with TMRE confirms an age-independent transition to the higher bioenergetic capacity caused by DR.

Given that proteins such as Bcl-X_L_ and Bcl-X_S_ take part in the regulation of the apoptotic process through the mitochondria-mediated intrinsic pathways and carrying anti- and pro-apoptotic function correspondingly [86], we analyzed the levels of these proteins (Figure 4). It is clear that in adult and old organisms, apoptotic cell death provides a protective mechanism by selective elimination of senescent or preneoplastic cells that could negatively affect normal function and/or promote cell transformation [87]. This process becomes even more important considering the fact that senescent cells are characterized by an elevated apoptotic resistance and such cells need a stronger signal to be eliminated [88]. In some studies, DR was suggested to enhance apoptosis [89]. However, due to the duration of DR, increased apoptosis is difficult to detect. For instance, after 8 weeks of DR, the levels of cleaved caspase-3 were significantly lower in comparison with kidney samples of the AL group [90]. Long-term DR led to the suppression of apoptosis in aged kidneys measured by Bcl-2, Bax, and cleaved caspase-3 levels [91]. Basing on our data (Figure 4), we suggest that mitochondria with low Bcl-X_L_ level and high Bcl-X_S_ levels were eliminated more effectively during DR ultimately causing accumulation of cells with a higher content of anti-apoptotic and lower content of pro-apoptotic proteins.

Another important factor reflecting the state of mitochondria is the activity of deacetylase SIRT-3. This NAD+-dependent deacetylase is localized predominantly in mitochondria and is considered to be a mitochondrial stress sensor that can modulate the activity of several mitochondrial proteins involved in metabolism and oxidative stress regulatory pathways [42]. SIRT-3 is suggested to be one of the mediators of the protective effect of DR [92]. We observed that DR increased the levels of SIRT-3 but only in young rats (Figure 4) which could be one more mechanism that has been deteriorated by aging.

In our study, mitochondria biogenesis was evaluated measuring PGC-1α levels (Figure 4). PGC-1α is a transcriptional coactivator playing a major role in the regulation of mitochondrial biogenesis, peroxisomal biogenesis, and glucose and lipid metabolism [43]. In our study, in young rats, PGC-1α levels increased significantly after 4 weeks of DR, whereas kidney tissue of old rats did not demonstrate such changes. Despite the important role of PGC-1α in energy homeostasis, the link between elevated PGC-1α levels during DR and mitochondria biogenesis remains a debatable question [93]. On the one hand, some studies provided evidence for the mitochondrial adaptations to DR by mitochondria biogenesis. Such conclusions were based on measuring PGC-1α protein level and RNA expression, increased mitochondrial DNA, cytochrome c, and cytochrome oxidase subunit IV in cells [94, 95]. On the other hand, the majority of studies refuted this position and, on the contrary, detected lower or unchanged activities of various mitochondrial enzymes [96]. Nevertheless, elevated PGC-1α level is considered to be associated with positive effects, including kidney tissue during aging [97].

Thus, in our study, after DR we observed some signs of improvement of the mitochondrial parameters both in young and old animals, in which the positive changes were not so pronounced (Figure 4). Elimination of mitochondria with low membrane potential is very important for the cells since dysfunctional mitochondria can be a source of pathological ROS, which are harmful to macromolecules and organelles [98]. Accumulation of damaged mitochondria as a result of the ineffective work of mitophagy leads to the progression of tissue aging [99]. A number of studies suppose the DR as a great approach for reducing oxidative stress in kidney tissue [30, 100, 101]. In our study, we confirmed these observations and revealed that DR even in kidney tissue of old rats was able to decrease tissue ROS levels and the levels of oxidative stress-modified proteins as well (Figure 6).

Of note, we detected a great difference in proliferative capacity in response to I/R in young and old rats (Figure 2). While in young animals the activation of proliferation in kidney tissue measured by PCNA levels after I/R increased 19 times, in kidney tissue of old rats proliferation rose only 4.5 times. This observation is consistent with earlier studies and supports the fact that regeneration capacity is deteriorated with aging [102, 103].

We also analyzed the influence of DR on kidney proliferation after I/R. On the one hand, there is an opinion that DR is able to activate stem cells in different tissues [104, 105]. It is believed that normal caloric intake increases the metabolic rate and damages in the stem cells which increases the rate of differentiation and reduction of self-renewal, functional, and regenerative capacity of stem cells. Under the restricted conditions, in the stem cells, the metabolic rate and damages are decreased which reduces the rate of differentiation and increases the self-renewal, functional, and regenerative capacity of the stem cells [106]. On the other hand, DR normally reduces the proliferation of cells due to inhibition of mTOR signaling [107], and activation of stem cells may occur through other pathways. In our study, we did not observe any further activation of kidney cells during I/R in response to DR (Figure 2). It remains an interesting issue for further studies and might be associated with the complexity of kidney regenerative mechanisms [108].

## CONCLUSIONS

Thus, in this study, we demonstrate that relatively short-term DR is beneficial even in old rats, but does not affect all mechanisms activated in young animals. Nonetheless, despite all observed protective signs, DR was not able to ameliorate kidney injury caused by I/R in old rats. It is possible, that molecular, structural, and functional deleterious changes occurring during aging do not allow DR to afford effective kidney protection. There are also some comorbidities accompanying aging including excessive weight, metabolic disorders, changes in hormone status, and others. Probably, old organisms require a more prolonged DR period to achieve a significant nephroprotective effect.

## MATERIALS AND METHODS

### Animals

Experiments were performed on male young (3-4-month-old, 300-400 g) and old (21-24-month-old, 550-700 g) outbred Wistar rats. Rats were used according to animal protocols evaluated and approved by the animal ethics committee of A.N. Belozersky Institute of Physico-Chemical Biology: Protocol 3/19 from March 18, 2019. All procedures were in accordance with the Federation of Laboratory Animal Science Associations (FELASA) guidelines. Rats were randomly divided into the following experimental groups with 5-6 animals in each.

### Dietary protocol

The amount of food consumed AL was approximately 25 gram/day for young rats and 32 gram/day for old rats, as measured by weighing the remaining food for two weeks. In young rats, DR was performed for 4 weeks by limiting the amount of food by 35% of the daily intake. In old rats, we applied 35% DR protocol lasting for 8 weeks, since in previous study we failed to reach an efficient nephroprotection by imposing old rats to 35% DR for 4 weeks [30]. Food was administered once daily at 1:00 pm. For all groups, free access to water was implemented. Weekly, every rat was weighed for monitoring changes in body mass.

### Kidney I/R protocol

Rats were anesthetized with chloralhydrate (300 mg/kg, i.p.) and subjected to 40-minutes warm ischemia of the left kidney as previously described [109]. Briefly, a renal vascular bundle was occluded with a microvascular clip for 40 min. Nephrectomy of the right kidney was performed simultaneously with ischemia of the left one; tissue samples were frozen in liquid nitrogen immediately after obtaining. After 40 min of ischemia, circulation was restored by removing the microvascular clip. The lack of blood flow during ischemia and its restoration during reperfusion were assessed visually. During the surgery procedure, the body temperature of the rats was maintained at 37 ± 0.5 °C. To confirm AKI, blood samples were taken 48 hours after I/R from the carotid artery to determine BUN and SCr. We analyzed urine samples taken 24 h after I/R for NGAL using western blotting.

### Western blotting

As control samples for western blotting, right kidneys were used, those were removed during nephrectomy in kidney I/R protocol. Such procedure allowed us to compare kidney samples from the same rats before and after exposure to I/R. Rats were sacrificed 48 hours after I/R; all kidneys were frozen in liquid nitrogen. After defrosting, tissue was homogenized with a glass-teflon homogenizer in a PBS buffer containing 10 mM phenylmethylsulfonylfluoride at 4°C. The homogenate was centrifuged at 3000 rpm for 3 min, the supernatant was mixed with 4x sample buffer containing 10% 2-mercaptoethanol, and boiled for 5 min. Kidney samples were loaded onto 15% Tris-glycine polyacrylamide gels (10 μg protein/lane). After electrophoresis, gels were transferred onto PVDF membranes (Amersham Pharmacia Biotech, UK). Membranes were blocked with 5% blocking agent (Amersham Pharmacia Biotech, RPN2125V, UK) in TBS containing 0.05% Tween-20 and subsequently incubated with primary antibodies: anti-α-smooth muscle actin rabbit 1:1000 (Abcam, UK), anti-PCNA 1:1000 rabbit (Cell Signaling, USA), anti-LC3 A/B rabbit 1:1000 (Cell Signaling, USA), anti-LAMP-1 rabbit 1:1000 (Abcam, UK), anti-p62/SQSTM1 rabbit 1:1000 (Cell Signaling, USA), anti-Bcl-X rabbit 1:1000 (Cell Signaling, USA), anti-SIRT-3 rabbit 1:1000 (Cell Signaling, USA), anti-PGC-1α rabbit 1:1000 (Invitrogen, Thermo Fisher Scientific, USA), anti-β-actin mouse 1:2000 (Sigma, USA). Membranes were then incubated with secondary antibodies: anti-rabbit IgG or anti-mouse IgG conjugated with horseradish peroxidase 1:7500 (Jackson ImmunoResearch, USA) and probed with Advansta Western Bright ECL kit (Advansta, USA). Detection was performed by V3 Western Blot Imager (BioRad, USA). Protein concentration was measured by bicinchoninic acid assay (Sigma, USA). Carbonylated proteins were measured using the OxyBlot kit according to the manufacturer’s instructions (S7150 OxyBlot Protein Oxidation Detection Kit, Millipore, USA).

Urine samples were centrifuged at 10 000× g, mixed with sample buffer, and loaded onto 15% Tris-glycine polyacrylamide gels (20 μl/lane). Electrophoresis, transfer, and blocking were performed as described above. Urine NGAL level was analyzed with anti-NGAL rabbit 1:1000 antibodies (Abcam, UK).

### Biochemical analysis of serum

Samples of the whole blood from intact rats and 48 h after I/R were taken from the carotid artery. After 15 min at the room temperature, the clot was removed by spinning at 2 000x g for 5 minutes in a centrifuge at 2-4°C. The resulting serum was frozen and later analyzed for SCr, BUN, triglycerides, and cholesterol concentrations. Biochemical parameters were analyzed using the AU480 Chemistry System (Beckman Coulter, USA).

### ELISA

Adiponectin levels were measured in serum by Mini Samples ELISA Kit for adiponectin according to the manufacturer’s instructions (Cloud-Clone Corp., CPR). Samples of serum were obtained as described above.

### Vital kidney slices imaging

Kidneys were excised and placed in the incubation medium (DMEM/F12 without sodium bicarbonate) to wash out the blood. Then, 100-150 μm thick sections through the cortical zone of the kidneys were made. Vital tissue slices were washed using the incubation medium (all procedures and incubation were done at 25°C) and loaded for 30 minutes with following dyes: 1 μM LysoTracker Green, 1 μM MitoTracker Green, 200 nM TMRE, 10 μM DCF (ThermoFisher Scientific, USA). For the detection of lipid peroxidation, we used Image-iT™ Lipid Peroxidation Kit according to the manufacturer’s protocols (ThermoFisher Scientific, USA). Kidney slices were imaged with LSM510 inverted confocal microscope (Carl Zeiss, Germany) with a pinhole at 150 μm. Slices loaded with LysoTracker Green, MitoTracker Green, and DCF were analyzed at emission 500-530 nm (excitation, 488 nm), and TMRE was evaluated at emission >560 nm (excitation at 543 nm). Image-iT™ Lipid Peroxidation Kit required to have the ratio of green to red fluorescence intensities, so the analysis was performed using both lasers consistently. Images were processed by ImageJ software (NIH, USA) [110], to quantify the mean intensity of fluorescence.

### Transmission electron microscopy

The cortical area of the kidney was cut into pieces approximately 1 mm 3 (upper case) in volume and fixed with 2.5% glutaraldehyde (Sigma, USA) in the Sorensen phosphate buffer (pH 7.4). Fixed samples were postfixed with 1% OsO_4_ in PBS, followed by dehydration in ascending acetone concentrations, stained with 1% uranyl acetate in 70% acetone during dehydration and embedded in EPON™–Araldite mixture resin. After polymerization, 80 nm thick sections were made using the LKB Nova ultramicrotome (Sweden). Sections were collected on a carbon-coated Cu grid, stained with lead citrate, according to Reynolds [111] and viewed with the electron microscope JEM-1400 (“JEOL”, Japan) at a 100 kV accelerating voltage.

### Statistics

Values are presented as mean ± SEM. Comparisons between groups were made using Mann-Whitney U-test. Data was analyzed in Microsoft Excel.

## Supplementary Material

Supplementary Figures
