# Supplementary material for: Resemblance and differences in dietary restriction nephroprotective mechanisms in young and old rats
Source: Aging (Albany NY). 2020 Sep 24;12(18):18693–715. doi: 10.18632/aging.103960 (PMC7585108; doi:10.18632/aging.103960)
Supplement: Supplementary Figures [file aging-12-103960-s001..pdf]

SUPPLEMENTARY FIGURES

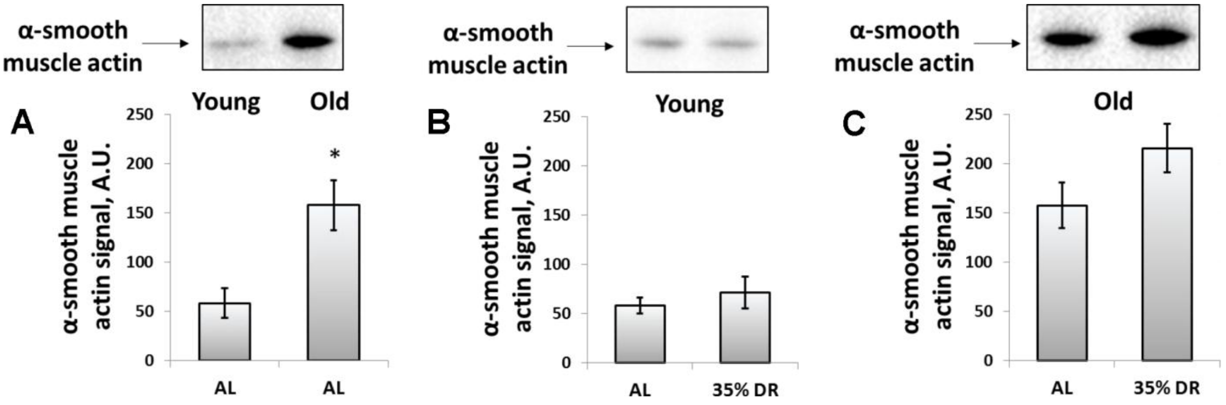

Supplementary Figure 1. Fibrosis in kidney tissue. (**A**) The comparison of α-smooth muscle actin levels in young and old rats; (**B**) Levels of α-smooth muscle actin in kidney tissue of young rats on AL or 35% DR diet; (**C**) Levels of α-smooth muscle actin in kidney tissue of old rats on AL or 35% DR diet. *$p < 0.05$ compared to young AL-group. For young AL rats n=5, for young DR rats n=6, for old AL rats n=6, for old DR rats n=5. AL, ad libitum, DR, dietary restriction.

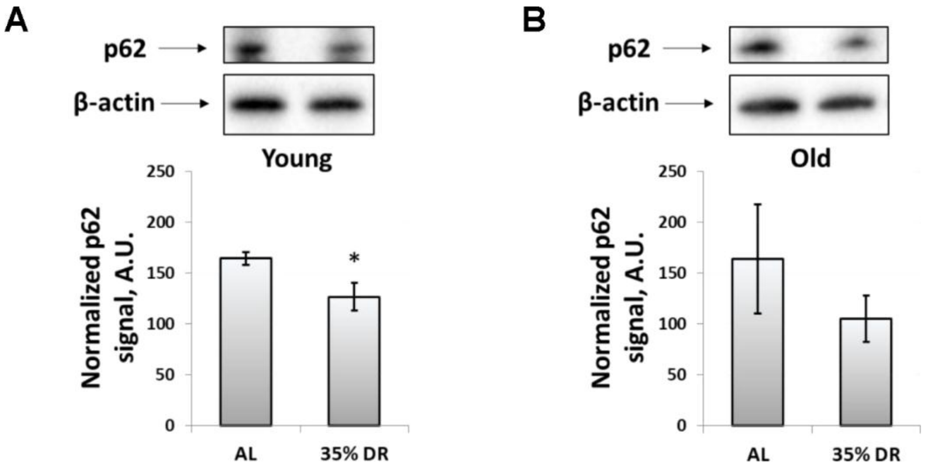

Supplementary Figure 2. The content of p62/SQSTM1 in kidney homogenates. (**A**) Level of p62/SQSTM1 in kidney tissue of young rats on AL or 35% DR diet; (**B**) Level of p62/SQSTM1 in kidney tissue of old rats on AL or 35% DR diet. *$p < 0.05$ compared to young AL-group. For young AL rats n=5, for young DR rats n=6, for old AL rats n=6, for old DR rats n=5. AL, ad libitum, DR, dietary restriction.

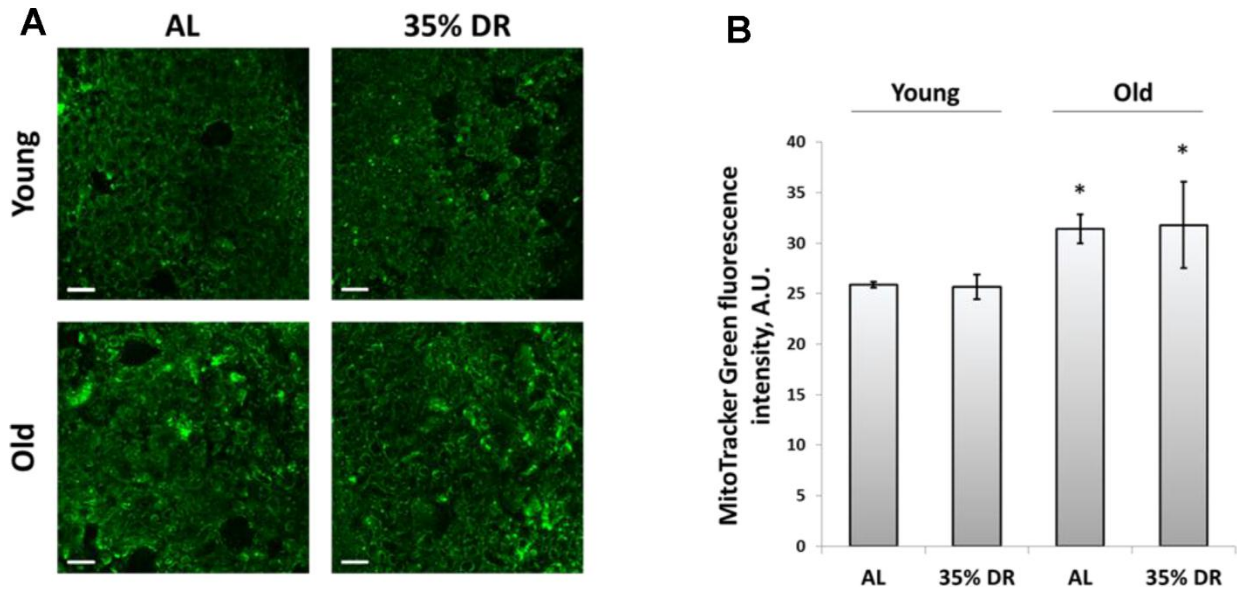

Supplementary Figure 3. Evaluation of MitoTracker Green staining. (**A**) Confocal microscopy of vital kidney slices loaded with mitochondrial dye MitoTracker Green. Scale bar, 100 µm; (**B**) Quantification of mean MitoTracker Green fluorescence intensity. *$p < 0.05$ compared to young AL-group. For young AL rats n=5, for young DR rats n=6, for old AL rats n=6, for old DR rats n=5. AL, ad libitum, DR, dietary restriction.
